# Associations of Milk Consumption and Vitamin B_2_ and Β_12_ Derived from Milk with Fitness, Anthropometric and Biochemical Indices in Children. The Healthy Growth Study

**DOI:** 10.3390/nu8100634

**Published:** 2016-10-13

**Authors:** George Moschonis, Ellen G. H. M. van den Heuvel, Christina Mavrogianni, Cécile M. Singh-Povel, Michalis Leotsinidis, Yannis Manios

**Affiliations:** 1 Department of Nutrition and Dietetics, Harokopio University, 70 El Venizelou Avenue, Kallithea, 17671 Athens, Greece; gmoschi@hua.gr (G.M.); cmavrog@hua.gr (C.M.); 2 EnviNHealth S.A., Platonos 34, Moschato, 18345 Athens, Greece; 3 FrieslandCampina, Stationsplein 4, Post Box 1551, 3800 BN Amersfoort, The Netherlands; ellen.vandenheuvel@frieslandcampina.com (E.G.H.M.v.d.H.); Cecile.Singh-Povel@frieslandcampina.com (C.M.S.-P.); 4 Department of Public Health, School of Medicine, University of Patras, 26504 Patras, Greece; mleon@med.upatras.gr

**Keywords:** milk, vitamin B_2_, vitamin B_12_, zinc, homocysteine, fitness, serum lipids, inflammation, children

## Abstract

The benefits of dairy consumption seem to extend beyond its significant contribution to ensuring nutrient intake adequacy as indicated by the favourable associations with several health outcomes reported by different studies. The aims of the present study were to examine the associations of milk consumption with fitness, anthropometric and biochemical indices in children and further explore whether the observed associations are attributed to vitamins B_2_ and B_12_ derived from milk. A representative subsample of 600 children aged 9–13 years participating in the Healthy Growth Study was examined. Data were collected on children’s dietary intake, using 24 h recalls, as well as on fitness, anthropometric and biochemical indices. Regression analyses were performed for investigating the research hypothesis, adjusting for potential confounders and for B-vitamin status indices (i.e., plasma riboflavin, methylmalonic acid and total homocysteine concentrations), dietary calcium intake and plasma zinc concentrations that could possibly act as effect modifiers. Milk consumption was positively associated with the number of stages performed in the endurance run test (ERT) (β = 0.10; *p* = 0.017) and negatively with body mass index (BMI) (β = −0.10; *p* = 0.014), after adjusting for several potential confounders and effect modifiers. Dietary intakes of vitamin B_2_ and B_12_ derived from milk were also positively associated with the number of ERT stages (β = 0.10; *p* = 0.015 and β = 0.10; *p* = 0.014 respectively). In conclusion, higher intake of milk as well as vitamin B_2_ and B_12_ derived from milk were independently associated with higher cardiorespiratory fitness in Greek preadolescents. The key roles of these B-vitamins in substrate oxidation, energy production, haemoglobin synthesis and erythropoiesis could provide a basis for interpreting these associations. However, further research is needed to confirm this potential interpretation.

## 1. Introduction

Milk and other dairy products (cheese and yogurt) are natural, rich sources of a wide range of essential nutrients [1,2]. In addition to their high protein content, daily consumption of dairy products contributes considerably to the overall diet quality and to the dietary intake adequacy of both vitamins (i.e., vitamins B_2_ and B_12_) and minerals (i.e., calcium, zinc, etc.) [1,2]. For these reasons, dairy products are an integral part of numerous national dietary guidelines, with the majority of these guidelines proposing consumption of 2–3 servings per day [3,4,5].

The important role of dairy in ensuring nutrient intake adequacy and sufficient concentrations of nutrient status blood indices was recently reported for the population of schoolchildren examined in the present study [2,6]. These recent findings showed that dairy consumption contributes to 63.2% of dietary calcium intake and 28.1% of total dietary zinc intake, as well as to 47.2%, 42.9% and 17.3% of the total dietary intakes of vitamin B_12_, B_2_ and B_6_ respectively [2]. The same data also showed that more that 50% of dairy consumption by the same population of Greek schoolchildren is attributed to milk intake, which subsequently contributes to 36.6%, 13.3%, 28.4%, 26.6% and 11.9% of the total dietary intakes of calcium, zinc, vitamin B_12_, B_2_ and B_6_ respectively [6]. Furthermore, it was confirmed that dietary vitamin B_2_ intake from milk was positively associated with plasma riboflavin concentration, i.e., a marker of vitamin B_2_ status [6]. In addition, dietary intakes of vitamin B_6_ and B_12_ from milk were associated with lower plasma concentrations of total homocysteine (tHcy) [6], a potential cardio metabolic risk factor that increases in states of poor B-vitamin status.

However, the benefits of dairy and milk in particular, seem to extend beyond significant contribution to the total dietary intake of several essential nutrients. In this context, there are several studies underscoring the benefits of total dairy and milk consumption on several health indices. More specifically, different meta-analyses have reported neutral or favourable associations of total dairy and milk consumption with body weight and fat mass levels [7,8], glycaemic and lipidemic profile [9,10], blood pressure and cardiorespiratory fitness indices in all age groups [11], as well as with optimal skeletal growth and development in children and adolescents [3]. Several nutrients, naturally present in milk and other dairy products, seem to have a key role in interpreting the favourable associations. In this regard, all dairy products contain all the essential amino acids and support muscle protein synthesis [12]. In addition, the role of dairy calcium on faecal fat excretion, appetite control, fat mobilization and oxidation [13], has been proposed as another basis for explaining the inverse associations observed between dairy intake and adiposity indices. Furthermore, numerous metabolic and cellular functions are mediated by zinc (e.g., immune function, protein synthesis, wound healing, DNA synthesis, cell division) and vitamins B_2_ and B_12_ (e.g., energy production, fatty acid synthesis and oxidation, mitochondrial function, haemoglobin synthesis), in the total dietary intake of which dairy products, especially milk, has been reported to considerably contribute in children [2]. However, research has not examined whether the health benefits associated with dairy consumption can be attributed to the vitamins and minerals naturally present in dairy products.

Due to the scarcity of relevant data, particularly in children and adolescents, the primary objective of the present study was to examine the associations of milk consumption with several fitness, anthropometric and biochemical indices of health status in schoolchildren aged 9–13 years, considering that more than 50% of dairy consumption by Greek schoolchildren is attributed to milk. A secondary objective was to explore whether these associations were probably attributed to specific nutrients for which milk consumption considerably contributes to their total dietary intakes, such as vitamins B_2_ and B_12_. Biomarkers of B-vitamins and zinc plasma concentration were used in the present study as possible effect modifiers of the aforementioned associations, since in many cases biomarkers adequately reflect dietary intakes [14,15].

## 2. Materials and Methods

### 2.1. Study Design and Study Population

The “Healthy Growth Study” was a cross-sectional study, the pilot phase of which was initiated in May 2007. Approval to conduct the study was granted by the Greek Ministry of National Education and the Ethical Committee of Harokopio University of Athens. The population under study comprised of schoolchildren aged 9–13 years attending the 5th and 6th grades from primary schools located in municipalities within the wider region of Athens.

The sampling of municipalities and schools in the “Healthy Growth Study” study was random, multistage and stratified by parental educational level and the total population of 9–13 year-old students, thus yielding a representative sample of primary schoolchildren. An appropriate number of schools were randomly selected from the participating municipalities in relation to the population of schoolchildren registered in the 5th and 6th grade in each municipality, based on data obtained from the Greek Ministry of Education. In total, for 2472 out of 4145 children (Response rate ~60%), a consent form signed by the parents or guardians was collected. Of these 2472 children, a representative subsample of 600 children was used to examine the current research hypothesis, for which data were already available from previous analyses on B-vitamin status markers. The selected subsample of 600 children had similar characteristics with the remaining subsample of 1872 children from the total study population, since there were no statistically significant differences observed in demographic, anthropometrical and clinical indices between the two samples [6]. More information on the sampling procedures followed is presented in details elsewhere [16].

### 2.2. Dietary Intake

Dietary intake data were obtained by trained dieticians and nutritionists via three 24-h recall morning interviews (i.e., in two consecutive weekdays and one weekend day) conducted with children at school-site. More information on the procedures followed to record and assess dietary intake is provided elsewhere [2,17]. Information on supplement usage was also collected as part of the 24 h recall interviews. Nevertheless, as supplement use has been reported to be very limited in Southern European countries including Greece [18], especially by children, supplements contribute only marginally to the total dietary intake of nutrients. Dietary intake data, in terms of food, energy and nutrient intakes, were analysed using the Nutritionist V diet analysis software (version 2.1, 1999, First Databank, San Bruno, CA, USA), which was extensively amended to include traditional Greek dishes and recipes [19]. Furthermore, the database was updated with nutritional information of processed foods publicly available (i.e., from food labels, products’ websites etc.).

The distribution of usual intakes of the B-vitamins under study was estimated by using the National Research Council method, which attempts to remove the effects of day-to-day (within subject) and subject-by-subject (between-subject) variability in dietary intakes [20]. To check for underreporting, the ratio of reported energy intake (EI) and the predicted basal metabolic rate was used. The basal metabolic rate (BMR) was estimated according to Schofield’s equations [21], taking into account age, sex and body weight. For the identification of those study participants that under-reported their energy intake, the age and sex-specific EI:BMR ratio cut-off points proposed by Goldberg et al. [22] were used. The identified under-reporters were not included in the subsample of 600 children examined in the current study.

### 2.3. Measurement of Haematological and Biochemical Indices

Blood samples were obtained for biochemical screening tests between 08.30 and 10.30 after a 12-h overnight fast. Reminders were distributed the previous day to both parents and children to ensure compliance with fasting. Professional staff performed venipuncture to obtain a maximum of 25 mL blood. Blood was collected in test tubes with or without ethylenediaminetetraacetic acid (EDTA) as anticoagulant. A part of the EDTA-containing whole blood was analysed on the same day of collection in a CELL-DYN haematological autoanalyser (Abbott Diagnostics, Abbott Park, IL, USA) for the determination of white blood cell (WBC) count and lymphocytes (% of WBC). The rest of the collected blood, with and without anticoagulant, was centrifuged at 3000 rpm for 15 min to isolate plasma and serum, respectively. The collected plasma and serum samples were pipetted into aliquots of 0.5 mL that were stored at −80 °C.

Serum was used to measure the concentrations of lipids, iron and inflammation status indices. More specifically, the concentrations of total cholesterol (TC), high-density lipoprotein cholesterol (HDLC) and triglycerides were measured using a colorimetric assay (Roche Diagnostics SA, Basel, Switzerland). Low-density lipoprotein cholesterol (LDLC) was calculated as: LDLC = TC − HDLC + triglycerides/5 [23]. The concentration of serum iron and total iron binding capacity (TIBC) were determined by colorimetric assays (Roche Diagnostics SA, Basel, Switzerland). Transferrin saturation (TSAT) was calculated by dividing serum iron by TIBC and multiplying by 100. Serum ferritin was measured by using a chemiluminescence immunoassay (Siemens Healthcare Diagnostics, Tarrytown, NY, USA). C-reactive protein (CRP) and interleukin 6 (IL-6) were measured with an enzyme-linked immunosorbent assay (ELISA) (R and D Systems, Minneapolis, MN, USA).

Plasma was used to measure the concentrations of total homocysteine (tHcy) as well as B-vitamin and zinc status indices. The concentrations of tHcy and riboflavin were measured using HPLC with fluorescence detection [24,25], while methylmalonic acid (MMA) was determined using liquid chromatography-tandem mass spectrometry (LC-MS/MS) with negative electrospray ionization (ESI) and multiple reaction monitoring (MRM) mode at mass system [26]. The main reagents (standards, controls and columns) for these plasma analyses were supplied by Chromsystems (Chromsystems Instruments and Chemicals GmbH, Grafelfing, Germany). According to the quality control measures conducted before performing the measurements of the plasma concentrations of the aforementioned indices, the inter-assay coefficients of variation were 3.2%–3.6% for plasma MMA levels, 2.9%–3.1% for plasma tHcy levels and <4.0% for plasma vitamin B_2_ levels. For the determination of zinc, plasma was diluted with deionized water, with a ratio of 1:5. Working standard solutions were prepared by dilution of the stock standards with 5% (*v*/*v*) glycerol and determination was performed by atomic absorption spectrophotometer (Shimadzu AA6300) in flame mode. Recoveries of spiked samples ranged between 95% and 105% while the intra- and inter-day variation was less than 10%. The laboratory of Public Health, in which the analyses were conducted, is accredited according to International Organization for Standardization (ISO) 17025 by the Hellenic Accreditation System (ESYD) for metal analyses.

### 2.4. Cardio-Respiratory Fitness and Muscle (Handgrip) Strength

Cardiorespiratory fitness was estimated indirectly according to children’s performance in the endurance 20-m shuttle run test (ERT). The ERT is a field test included in the European battery of physical fitness tests and recommended by the Committee of Experts on Sports Research [27]. Based on the test’s instructions, participants start running at a speed of 8.5 km/h while speed is gradually increasing in stages. Participants shuttled between two lines placed 20 m apart, at a pace dictated by a sound signal on an audiotape, which gets progressively faster (by 0.5 km/h every minute). Each stage of the test is made up of several shuttle runs and the score of the participant is the half-stage completed before the child drops out (thus, scores can be 0, 0.5, 1, 1.5, 2, etc.). The higher the ERT score, the better the cardiorespiratory fitness. Prior to the test, all children received clear and comprehensible instructions on rules and procedures, while during the test they were verbally encouraged by the researchers to reach their maximal number of laps. The ERT is recommended for large groups of children, since it is reliable, valid, non-invasive, and requires limited facilities [28]. Handgrip strength was measured three times on each side, alternating between right and left hands, using a portable Takei handgrip dynamometer (Takei Scientific Instruments Co. Ltd., Tokyo, Japan). Participants were given standardized encouragement to squeeze the dynamometer as hard as possible. The dynamometer was calibrated at the start of the study. The mean handgrip strength was calculated in each hand.

### 2.5. Anthropometric Indices

The protocol and equipment used for anthropometric measurements were the same in all schools. Body weight was measured to the nearest 10 g using a Seca digital scale (Seca Alpha, Model 770, Hamburg, Germany) in the minimum clothing possible. Height was measured to the nearest 0.1 cm using a commercial stadiometer (Leicester Height Measure, Invicta Plastics Ltd., Oadby, UK) with participants barefoot, their shoulders in a relaxed position, their arms hanging freely and their head aligned in Frankfort plane. Weight and height were converted to body mass index (BMI) using Quetelet’s equation (i.e., weight (kg)/height^2^ (m^2^)).

### 2.6. Physical Activity Levels

Physical activity was objectively measured using step counters and was used as one of the covariates in the current analyses. More specifically, children were provided with and instructed to wear a waist-mounted pedometer (Yamax SW-200 Digiwalker, Tokyo, Japan) for one week, i.e., from Monday to Sunday. Detailed information for the procedures followed for the recording of steps is provided elsewhere [29].

### 2.7. Statistical Analyses

Normality of the distribution of continuous variables was analysed using the Kolmogorov–Smirnov test. Non-normally distributed continuous variables were logarithmically transformed prior to any statistical analysis. Normally distributed continuous variables were displayed as the mean value ± standard deviation (sd), while non-normally distributed ones were displayed as the median values and interquartile range. Differences in mean or median values of continuous variables were examined using the Student’s *t*-test or the Mann–Whitney test in the case of normally or non-normally distributed variables, respectively. Regression analyses were performed for testing the associations between milk consumption (independent variable) and certain health indices (each health index was used as the dependent variable in the regression models tested), initially adjusting for age and sex (model 1) and for a wide range of other potential and relevant confounders, according to current knowledge and evidence (model 2). The use of these variables as confounders in the regression models was based on existing knowledge regarding the association of the potential confounder with the dependent and/or the independent variable in each regression model that could potentially affect their relationship, i.e., by strengthening or weakening it. In this regard, additional adjustments were made for B-vitamin status indices (model 3) as well as for dietary calcium intake and plasma zinc concentrations (model 4) that could possibly act as effect modifiers in the associations of milk consumption with health indices. The rationale of adjusting for plasma B-vitamins and zinc concentrations as well as dietary calcium intake in models 3 or 4 was based on the known role of these nutrients in the physiology related to each one of the examined health indices. In order to further investigate whether the observed associations between milk consumption and health indices are mediated by the dietary intake of vitamin B_2_ or vitamin B_12_ derived from milk, additional regression analyses were performed. In these regression analyses, adjustments were initially made for age, sex and a wide range of other relevant and potential confounders (model 1), while additional adjustments were also made for B-vitamin and zinc status indices (model 2), so as to examine the possible role of these variables as effect modifiers. All variables used as potential confounders or effect modifiers in each regression model are listed at the footnotes of Table 2 and Table 3. All statistical analyses were performed with the IBM SPSS Statistics version 21.0 (SPSS Inc., Dallas, TX, USA), all reported *p*-values were based on two-sided tests and the level of statistical significance was set at *p* < 0.05.

## 3. Results

Table 1 presents the descriptive characteristics of the study participants and the differences between boys and girls. Several significant differences were observed between the two sexes in dietary intake indices, with boys reporting higher dietary intakes of energy, total fat, monounsaturated and saturated fat, cholesterol, protein, carbohydrate, fibre, calcium, folate, vitamins B_2_, B_6_ and B_12_ and milk consumption compared to girls (*p* ≤ 0.01). Regarding biochemical indices, serum ferritin, serum CRP and plasma zinc concentrations were higher in boys in comparison to girls (*p* ≤ 0.001), while girls had higher concentrations of serum triglycerides compared to boys (*p* = 0.001). As far as fitness indices were concerned, boys were found to perform better in the endurance 20 m shuttle run test (ERT stages) and to have higher handgrip strength in both arms compared to their female counterparts (*p* < 0.001). Furthermore, boys recorded, on average, more daily steps compared to girls. Considering all aforementioned statistically significant differences between boys and girls, adjustments for sex were made in all regression models performed to examine the research hypothesis. No other statistically significant differences were observed between sexes.

Table 2 summarizes the associations of children’s milk consumption (in mL/day) with certain biochemical, fitness and anthropometrical indices in different regression models where gradual adjustments were made for potential confounders (models 1 and 2) as well as for effect modifiers (models 3 and 4). Milk consumption was negatively associated with serum triglycerides (β = −0.11; *p* = 0.010), ferritin (β = −0.08; *p* = 0.042) and CRP (β = −0.1; *p* = 0.013) concentrations but positively with HDLC (β = 0.11; *p* = 0.007) concentrations after adjusting for age and sex (model 1). Nevertheless, these associations became statistically non-significant when adjustments for other potential confounders were also performed (models 2 and 3). However, milk consumption remained significantly and positively associated with the number of stages (β = 0.09; *p* = 0.017) performed in the ERT, even after several adjustments for potential confounders (models 1 and 2) or effect modifiers (model 3) were made. In addition, milk consumption remained significantly and negatively associated with BMI (β = −0.10; *p* = 0.014), even after controlling for a wide range of confounders (model 2), and lost statistical significance when also controlling for B-vitamins status (model 3) but regained statistical significance when adjustments were additionally made for dietary calcium intake (model 4).

Table 3 displays the associations of children’s dietary intake of vitamin B_2_ and B_12_ derived from milk with biochemical, fitness and anthropometrical indices, after gradually adjusting for a number potential confounders (model 1) and possible effect modifiers (model 2). Dietary intake of vitamin B_2_ derived from milk remained positively and significantly associated with serum HDLC (β = 0.08; *p* = 0.041), even after several adjustments were made. Furthermore, the association between dietary intake of vitamin B_12_ derived from milk and serum ferritin concentrations became statistically significant (β = −0.09; *p* = 0.029) when adjustments were made for B-vitamin status indices in model 2, while no significant association was observed in model 1. The dietary intakes of vitamin B_2_ and B_12_ derived from milk remained significantly associated with the number of stages performed in the ERT (β = 0.10; *p* = 0.015 and β = 0.10; *p* = 0.014 for vitamin B_2_ and B_12_ respectively) after adjusting for several potential confounders (model 1) and possible effect modifiers (model 2). No other statistically significant associations were observed.

**Table 2:** Regression models testing the associations of milk intake with biochemical, fitness and anthropometrical indices of health status after controlling for potential relevant confounders and possible effect modifiers.

Dependent Variable(s)	Independent Variable: Milk Intake (mL/Day)							
	Model 1		Model 2		Model 3		Model 4	
	β	*p*-Value	β	*p*-Value	β	*p*-Value	β	*p*-Value
**Biochemical indices (serum lipids)**								
Total cholesterol (mmol/L)	−0.02	0.593	−0.02	0.624	−0.02	0.635	-	-
Triglycerides (mmol/L)	−0.11	**0.010**	−0.07	0.085	−0.07	0.095	-	-
HDL cholesterol (mmol/L)	0.11	**0.007**	0.08	0.055	0.07	0.064	-	-
LDL cholesterol (mmol/L)	−0.07	0.085	−0.06	0.184	−0.05	0.198	-	-
**Biochemical indices (iron status)**								
Ferritin (pmol/L)	−0.08	**0.042**	−0.05	0.196	−0.07	0.080	-	-
Transferrin saturation (%)	0.02	0.632	−0.01	0.835	−0.02	0.572	-	-
**Biochemical indices (inflammation markers)**								
White Blood cells (10^9^/L)	−0.21	0.610	−0.02	0.685	−0.01	0.872	−0.02	0.670
Lymph cells (%)	0.20	0.631	0.17	0.681	0.01	0.778	0.01	0.750
CRP (nmol/L)	−0.10	**0.013**	−0.07	0.114	−0.07	0.115	−0.06	0.132
Interleukin-6 (pg/mL)	0.02	0.698	0.02	0.624	0.03	0.515	0.02	0.594
**Fitness indices**								
Endurance Run test Stages	0.16	**<0.001**	0.11	**0.008**	0.10	**0.017**	-	-
Right handgrip strength	−0.02	0.590	0.001	0.982	0.01	0.782	-	-
Left handgrip strength	−0.01	0.826	0.03	0.504	0.04	0.333	-	-
**Anthropometrical indices**								
Height (cm)	0.003	0.944	−0.01	0.818	0.01	0.892	0.02	0.601
Body Mass Index (kg/m^2^)	−0.13	**0.002**	−0.09	**0.038**	−0.07	0.111	−0.10	**0.014**

HDL: High-density lipoprotein; LDL: low-density lipoprotein; CRP: C-reactive protein. Model 1 was adjusted for age and sex; Model 2 was adjusted for age, sex, dietary energy intake and total steps per day in the case of all dependent variables. Adjustments were also made for dietary protein intake in the case of inflammation, fitness and anthropometrical indices; for dietary fibre and fat intake in the case of serum lipids, iron status, inflammation and anthropometrical indices; for dietary carbohydrate intake in the case of inflammation and anthropometrical indices; for Body Mass Index (BMI) in the case of serum lipids, iron status, inflammation and fitness indices; for dietary cholesterol intake in the case of serum lipids; for CRP and IL-6 in the case of iron status indices; and for hemoglobin levels in the case of fitness indices; Model 3 was adjusted for the same variables as in model 2 and for plasma riboflavin levels in the case of all dependent variables. Adjustments were also made for total plasma total homocysteine (tHcy) and methylmalonic acid (MMA) in the case of iron status, inflammation, fitness and anthropometrical indices; Model 4 was adjusted for the same variables as in model 3 and for zinc levels in the case of inflammation and anthropometrical indices; for dietary calcium intake in the case of BMI.

**Table 3:** Regression models testing the associations of vitamin B_2_ and vitamin B_12_ derived from milk with biochemical, fitness and anthropometrical indices of health status after controlling for potential relevant confounders and possible effect modifiers.

Dependent Variable(s):	Independent Variable: Vitamin B_2_ Derived from Milk (mg/Day)				Independent Variable: Vitamin B_12_ Derived from Milk (mg/Day)			
	Model 1		Model 2		Model 1		Model 2	
	β	*p*-Value	β	*p*-Value	β	*p*-Value	β	*p*-Value
**Biochemical indices (serum lipids)**								
Total cholesterol (mmol/L)	−0.02	0.600	−0.02	0.591	−0.02	0.542	−0.03	0.525
Triglycerides (mmol/L)	−0.08	0.056	−0.07	0.073	−0.08	0.067	−0.07	0.090
HDL cholesterol (mmol/L)	**0.12**	**0.006**	**0.08**	**0.041**	0.07	0.082	0.07	0.099
LDL cholesterol (mmol/L)	−0.07	0.081	−0.06	0.149	−0.07	0.092	−0.06	0.171
**Biochemical indices (iron status)**								
Ferritin (pmol/L)	−0.07	0.135	−0.08	0.057	−0.08	0.076	**−0.09**	**0.029**
Transferrin saturation (%)	−0.02	0.641	−0.04	0.297	0.02	0.717	−0.04	0.337
**Biochemical indices (inflammation markers)**								
White Blood cells (10^9^/L)	−0.006	0.891	−0.003	0.936	0.001	0.998	0.001	0.975
Lymph cells (%)	0.01	0.745	0.02	0.703	0.01	0.733	0.02	0.679
CRP (nmol/L)	−0.07	0.112	−0.08	0.080	−0.08	0.098	−0.08	0.085
Interleukin−6 (pg/mL)	0.02	0.546	0.02	0.610	0.03	0.477	0.02	0.568
**Fitness indices**								
Endurance Run test Stages	**0.11**	**0.006**	**0.10**	**0.015**	**0.12**	**0.006**	**0.10**	**0.014**
Right handgrip strength	0.01	0.884	0.02	0.702	0.01	0.789	0.02	0.627
Left handgrip strength	0.03	0.466	0.04	0.344	0.03	0.467	0.04	0.357
**Anthropometrical indices**								
Height (cm)	0.003	0.952	0.03	0.555	−0.004	0.920	0.02	0.632
Body Mass Index (kg/m^2^)	−0.07	0.114	−0.05	0.252	−0.07	0.082	−0.06	0.192

HDL: High-density lipoprotein; LDL: low-density lipoprotein; CRP: C-reactive protein. Model 1 was adjusted for age, sex, dietary energy intake, total number of steps per day in the case of all dependent variables. Adjustments were also made for dietary fat intake in the case of serum lipids, inflammation markers and anthropometrical indices; for dietary protein intake in the case of inflammation markers, fitness and anthropometrical indices; for dietary carbohydrates intake in the case of inflammation markers and anthropometrical indices; for dietary cholesterol intake in the case of serum lipids; dietary fibre intake in the case of serum lipids, iron status indices, inflammation markers and anthropometrical indices; for dietary iron intake in the case of iron status indices; for Body Mass Index (BMI) in the case of serum lipids, iron status indices, inflammation markers and fitness indices; for CRP and IL-6 in the case of iron status indices; and for haemoglobin levels in the case of fitness indices; Model 2 was adjusted for the same variables as in model 1 and for plasma riboflavin levels in the case of all dependent variables. Adjustments were also made for total plasma total homocysteine (tHcy) and for methylmalonic acid (MMA) in the case of iron status indices, inflammation markers, fitness and anthropometrical indices; and for plasma zinc levels in the case of inflammation markers and height.

## 4. Discussion

The current study highlighted a positive association between milk consumption with cardiorespiratory fitness, as indicated by children’s performance in the ERT. The evidence available in the literature regarding this specific association is very limited. In this regard, to our knowledge only the HELENA (Healthy Lifestyle in Europe by Nutrition in Adolescence) study has reported a strong positive association between intake of dairy products by European male and female adolescents and cardiorespiratory fitness levels [30]. Regarding prospective data, only one study reported a positive association of milk intake in childhood with physical performance in elderly and specifically at a mean age of 75.3 years [31]. As milk consumption has been previously reported to be part of a more favourable lifestyle pattern in children [32], increased physical activity levels recorded for children with higher milk consumption, could provide a basis for interpreting the positive association between milk intake and fitness levels observed in the present study. However, the association of milk consumption with cardiorespiratory fitness retained statistical significance even after gradually adjusting for a wide range of potential confounders, including dietary and physical activity indices among them, thus probably indicating an independent association.

Several metabolically active nutrients naturally present in milk could provide a basis for interpreting the possible independent association between milk consumption and cardiorespiratory fitness and, to an extent, the potential mechanisms underlying it. In this regard, this association could be interpreted by milk’s relatively high percent contribution to the total dietary intakes of vitamin B_2_ and B_12_ (i.e., 28.4% and 26.6% respectively) [6]. The key role of vitamin B_2_ in a diversity of redox reactions (particularly through the co-factors flavin mononucleotide (FMN) and flavin adenine dinucleotide (FAD) that act as electron carriers) is well established and highlights the importance of vitamin B_2_ in the human metabolism, substrate oxidation (e.g., *β* oxidation of fatty acids) and energy production [33,34,35]. Inadequate intake of vitamin B_2_ would therefore be expected to lead to metabolic disturbances, with subsequent functional implications including deterioration of physical performance. In addition to vitamin B_2_, vitamin B_12_ is also involved in energy utilization mainly via stimulation of mitochondrial function [36,37]. In addition, both vitamin B_2_ and B_12_ have a role in the process of haemoglobin synthesis and erythropoiesis [38,39], thus improving the capacity of oxygen transportation to tissues for energy production. Considering that the statistical significance of the associations between milk consumption, as well as vitamin B_2_ and B_12_ derived from milk and cardiorespiratory fitness was slightly attenuated after adjusting for B-vitamin status indices, the latter could possibly mediate these associations, most likely via the pathways mentioned above.

Regarding the negative and possibly independent association observed in the present study between milk consumption and BMI, dietary intakes of vitamin B_2_ and B_12_ derived from milk could also be implicated. More specifically, the rise in plasma concentrations of B-vitamins induced by milk consumption, as previously observed in the schoolchildren also examined in the present study [6], may mediate the favourable effect of milk on children’s weight status, possibly via the role of B-vitamins in substrate oxidation and energy production. Nevertheless, further research is needed to expand the knowledge on the possible role of B-vitamins derived from milk on children’s BMI. The high calcium content of milk could provide a more solid basis for another interpretation of the negative relation between milk consumption and BMI, especially when considering that this association regained statistical significance after adjusting for dietary calcium intake (i.e., model 4 in Table 2). In terms of suggested metabolic pathways, calcium has been proposed to decrease dietary energy intake (i.e., through the formation of calcium-fatty acids soaps and stimulation of satiety) and increase energy utilization (i.e., through increased fat oxidation) [40] thus favourably contributing to energy equilibrium. In this context, a meta-analysis has recently reported that an increase of calcium intake by ~800 mg/day would also favour an 11% increase in fat oxidation [41], while evidence from a second meta-analysis showed that for every ~1200 mg of calcium consumed daily, an excretion of ~5 g/day of fat (or 45 kcal/day) can be expected [42]. Considering that children in the present study reported a mean dietary calcium intake of ~1100 mg/day, with dairy and milk contributing to 63.2% and 36.6% (~700 or 420 mg/day) respectively of this intake, the negative association between milk intake and BMI observed in the present study could be supported, probably through the pathways mentioned above.

The present study revealed another positive association between dietary intake of vitamin B_2_ (as well as a tendency for vitamin B_12_) derived from milk and serum concentrations of HDLC even after several adjustments were made for dietary, physical activity, anthropometrical and B-vitamin status indices. Associations of borderline statistical significance (0.5 < *p* < 0.1) were also observed for serum concentrations of triglycerides, which tended to be associated with vitamin B_2_ and B_12_ derived from milk even after several adjustments were performed. Although riboflavin and flavoenzymes are known to be involved in the biosynthetic pathways of cholesterogenesis [43], additional research is still needed in order to elucidate this specific association.

Another interesting finding of the present study that warrants interpretation is the negative association observed between vitamin B_12_ derived from milk and serum ferritin concentrations following adjustments for B-vitamin status indices (i.e., model 2 in Table 3). One interpretation of this association could stem from the lowering in the blood levels of certain cardio-metabolic risk indices, attributed to several bioactive compounds found in milk, with vitamin B_12_ included among them. Considering the positive associations reported by previous studies between increased serum ferritin levels and the risk for atherogenesis [44,45,46,47], there might be a metabolic pathway inversely linking dietary vitamin B_12_ derived from milk with serum ferritin concentrations [48]. However, the above is only speculative and, as such, further research is needed to either provide a safe interpretation of the association observed in the present study between vitamin B_12_ derived from milk and serum ferritin concentrations or prove a random association.

The findings of the current study should be interpreted in light of its strengths and limitations. Adjustments for the effects of day-to-day (within-subject) and subject-by-subject (between-subject) variability to estimate usual dietary intakes, as well as exclusion of subjects that were under-reporting their food consumption could be considered as the strongest component of the methodological approach used in the present study. Regarding limitations, first and foremost, a cause-effect relationship cannot be identified due to the cross-sectional design of the current study. Secondly, although under-reporters were excluded in the present study, self-reporting of food intake data introduce bias to the dietary intake data. Thirdly, the use of plasma riboflavin concentrations to assess vitamin B_2_ status could be considered as another limitation of the present study, taking into account the light-sensitivity of flavonoids that could probably have affected the results of the present study regarding the reported associations between plasma riboflavin and health status indices. However, exposure of blood samples collected in the present study to (indoor) light was negligible, since the intermediate time between blood collection, processing and plasma storage was only a few minutes.

## 5. Conclusions

In conclusion, the present study showed that higher milk consumption was independently associated with higher cardiorespiratory fitness and lower BMI levels in Greek preadolescents. Dietary intakes of vitamin B_2_ and B_12_ intake derived from milk could provide a basis for interpreting these associations, especially when considering the key roles of these vitamins in substrate oxidation and energy production as well in haemoglobin synthesis and erythropoiesis. However, further research is needed in order to shed more light on the effects of the dietary intakes of vitamin B_2_ and B_12_, for which dairy products are among their major food sources, on several health status indices in children and adolescents.

## Figures and Tables

**Table 1:** Average age and levels of dietary intake, haematological, biochemical, fitness, anthropometrical and physical activity indices in the total sample and by sex.

	Total Sample (*n* = 600)	Boys (*n* = 296)	Girls (*n* = 304)	*p*-Value *
	Mean (SD)	Mean (SD)	Mean (SD)	
Age (years)	11.2 (0.7)	11.2 (0.6)	11.2 (0.7)	0.441
***Dietary intake indices***				
Energy intake (kcal/day)	1875 (556)	1987 (559)	1765 (532)	<0.001
Total fat intake (g/day)	85 (32)	90 (33)	80 (30)	<0.001
Monounsaturated fat intake (g/day)	39 (17)	42 (18)	37 (15)	<0.001
Polyunsaturated fat intake (g/day)	11 (9)	11 (7)	12 (11)	0.742
Saturated fat intake (g/day)	31 (12)	33 (13)	29 (11)	<0.001
Cholesterol intake (mg/day)	241 (120)	254 (123)	228 (115)	0.010
Protein intake (g/day)	72 (24)	77 (24)	67 (22)	<0.001
Carbohydrate intake (g/day)	215 (69)	227 (69)	203 (67)	<0.001
Fibre intake (g/day)	15 (8)	16 (8)	14 (8)	0.001
Calcium intake (mg/day)	1086 (409)	1147 (426)	1025 (382)	<0.001
Folate intake (μg/day)	249 (134)	269 (137)	230 (128)	<0.001
Vitamin B_2_ intake (mg/day)	1.8 (0.7)	1.8 (0.7)	1.6 (0.6)	<0.001
Vitamin B_6_ intake (mg/day)	1.8 (0.7)	1.9 (0.8)	1.7 (0.7)	<0.001
Vitamin B_12_ intake (μg/day)	4.8 (3.7)	5.2 (4.3)	4.3 (2.9)	0.001
Milk consumption (mL/day) ^†^	310 (181–488)	364 (244–516)	258 (129–440)	0.002
***Haematological indices***				
Haemoglobin (g/100 mL)	13.2 (0.86)	13.3 (0.85)	13.2 (0.87)	0.155
***Biochemical indices (serum)***				
***Lipids and lipoproteins***				
Total cholesterol (mmol/L)	4.33 (0.85)	4.37 (0.86)	4.29 (0.84)	0.238
Triglycerides (mmol/L)	0.71 (0.35)	0.66 (0.32)	0.75 (0.37)	0.001
HDL cholesterol (mmol/L)	1.56 (0.41)	1.59 (0.40)	1.53 (0.41)	0.098
LDL cholesterol (mmol/L)	2.45 (0.67)	2.48 (0.69)	2.41 (0.64)	0.202
***Inflammation markers***				
White Blood cells (10^9^/L)	6.62 (1.50)	6.56 (1.45)	6.67 (1.54)	0.348
Lymph cells (%)	37.9 (7.88)	38.3 (7.47)	37.6 (8.25)	0.242
CRP (nmol/L) ^†^	5.59 (2.04–14.3)	7.45 (2.37–17.4)	4.69 (1.70–11.9)	<0.001
Interleukin-6 (pg/mL)	1.14 (1.04)	1.14 (0.95)	1.13 (1.12)	0.959
***Zinc, iron and B vitamin status markers***				
Zinc (μmol/L)	12.4 (1.8)	12.6 (2.0)	12.1 (1.8)	<0.001
Iron (μmol/L)	15.1 (6.2)	14.8 (6.04)	15.3 (6.35)	0.257
Ferritin (pmol/L)	67.3 (40.5)	74.4 (45.6)	60.3 (33.5)	<0.001
Transferrin saturation (%)	25 (10)	25 (10)	25 (11)	0.999
Plasma riboflavin (μmol/L) ^†^	117.0 (82.5–169.6)	114.4 (85.1–156.3)	117.0 (79.8–183.5)	0.448
Plasma total homocysteine (μmol/L)	5.7 (1.8)	5.8 (1.7)	5.6 (1.9)	0.170
Plasma methylmalonic acid (nmol/L)	0.11 (0.06)	0.10 (0.06)	0.11 (0.06)	0.709
***Fitness indices***				
Endurance Run test Stages	2.3 (1.4)	2.7 (1.5)	1.9 (1.1)	<0.001
Right handgrip strength	19.5 (4.5)	20.3 (4.3)	18.8 (4.5)	<0.001
Left handgrip strength	18.6 (4.4)	19.2 (4.3)	18.0 (4.4)	<0.001
***Anthropometrical indices***				
Height (cm)	149.5 (7.7)	149.0 (7.5)	150.0 (7.9)	0.097
Body Mass Index (kg/m^2^)	20.5 (3.9)	20.7 (3.9)	20.4 (3.8)	0.334
***Physical activity indices***				
Steps (no per day)	13,105 (4777)	14,497 (4933)	11,749 (4205)	<0.001

SD: Standard deviation; HDL: high-density lipoprotein; LDL: low-density lipoprotein; CRP: C-reactive protein. * *p*-Values were derived from the Student’s *t*-test or the Mann–Whitney test whenever appropriate; ^†^ on-normally distributed variables displayed as median values (25th–75th quartile).
